# Elevational Distribution and Extinction Risk in Birds

**DOI:** 10.1371/journal.pone.0121849

**Published:** 2015-04-07

**Authors:** Rachel L. White, Peter M. Bennett

**Affiliations:** Durrell Institute of Conservation and Ecology, University of Kent, Canterbury, Kent, United Kingdom; University of Colorado, UNITED STATES

## Abstract

Mountainous regions are hotspots of terrestrial biodiversity. Unlike islands, which have been the focus of extensive research on extinction dynamics, fewer studies have examined mountain ranges even though they face increasing threats from human pressures – notably habitat conversion and climate change. Limits to the taxonomic and geographical extent and resolution of previously available information have precluded an explicit assessment of the relative role of elevational distribution in determining extinction risk. We use a new global species-level avian database to quantify the influence of elevational distribution (range, maximum and midpoint) on extinction risk in birds at the global scale. We also tested this relationship within biogeographic realms, higher taxonomic levels, and across phylogenetic contrasts. Potential confounding variables (i.e. phylogenetic, distributional, morphological, life history and niche breadth) were also tested and controlled for. We show that the three measures of elevational distribution are strong negative predictors of avian extinction risk, with elevational range comparable and complementary to that of geographical range size. Extinction risk was also found to be positively associated with body weight, development and adult survival, but negatively associated with reproduction and niche breadth. The robust and consistent findings from this study demonstrate the importance of elevational distribution as a key driver of variation in extinction dynamics in birds. Our results also highlight elevational distribution as a missing criterion in current schemes for quantifying extinction risk and setting species conservation priorities in birds. Further research is recommended to test for generality across non-avian taxa, which will require an advance in our knowledge of species’ current elevational ranges and increased efforts to digitise and centralise such data.

## Introduction

Recent global studies of the distribution of geographical range sizes across species have shown that major mountain chains, predominately within the tropics, are the richest areas for terrestrial biodiversity (e.g. [1–3]). For example, 89% of avian species richness hotspot regions are located in mountainous areas of mainland continents [1]. The reasons for this distribution are poorly understood; we have a limited understanding of the evolutionary and ecological factors that promote hotspots of avian diversity, including the relative importance of variation in speciation and extinction rates. This is partly due to the relative paucity of studies on mountain systems and elevational gradients, in comparison to the extensive literature on both island systems and latitudinal gradients in avian diversity (e.g. [3, 4–7]; however, see [8]). Furthermore, despite the importance of mountain ranges for global biodiversity conservation, we know little about the type, distribution and impact of the threats they face, which is essential for the effective prioritisation and implementation of conservation effort.

Here we investigate the relationship between extinction risk and elevational distribution in birds on a global scale. It has been widely proposed that climate change may cause extinctions in montane regions through the mechanism of upward elevational range shifts, with high-montane species being especially vulnerable to warming (e.g. [9–12]). This scenario predicts a positive relationship between extinction risk and elevation. Alternatively, species living in lowlands may face more direct human pressures, including habitat destruction and overexploitation [13–15]. Montane areas in comparison may be relatively unspoilt by direct anthropogenic activities, due to their inaccessibility and steep gradients [13–15]. This scenario predicts a negative association with elevation, with lowland species at greatest risk of extinction.

Small geographical range size is considered the single best predictor of threat of extinction in terrestrial species [13]. In comparison to the large literature exploring the relative roles of geographical range and latitudinal distribution on extinction risk across taxa (e.g. [1–2, 13, 16]), only a few studies have investigated species-level elevational distribution as a predictor of current extinction risk. The most studied taxa to date are birds (see S1 Table). Methodologically, the earliest avian studies were based on descriptive statistics and did not control for phylogeny [16–17], with more recent studies using regression-based approaches [18–19]. Existing avian studies that use species as the study unit are spatially and/or taxonomically restricted (e.g. [16–20]), with two using binary measures of both elevation and extinction risk [16–17]. Other studies have been global in extent, but utilise gridded data derived from satellite imagery to model potential elevational distribution [21–22], or use country as the study unit [23], rather than actual recorded elevational limits of each species. Elevational range is the most frequently studied elevational distribution extinction risk predictor. Both maximum elevation and elevational midpoint have been largely overlooked (but see [11 and 20]). Despite differences in aim, extent and methodology, several of these studies provide evidence for a negative relationship between avian extinction risk and elevational range [11, 17–19], although see [21–22]. Fewer studies found that lowland birds are more threatened with extinction than montane species [11, 16–17], although see [18, 20].

In addition, there have been some regional studies that have investigated range contractions—a component of extinction risk—by comparing historical and modern avian occupancy along altitudinal gradients, e.g. within the Grinnell Resurvey Project [24–26]. However, we lack an explicit global investigation of how avian elevational limits compare to well-established predictors of extinction risk including geographical range, latitude, and both life-history and ecological traits.

Understanding the global distribution of extinction risk is central to determining spatial priorities for the focus of conservation effort. The world is topographically complex, yet current models used to determine extinction risk assume species live in two dimensions, which is too simplistic, especially for taxa with high dispersal capabilities. Consequently, this study uses birds as a model system to investigate large-scale variation in extinction risk with respect to elevational gradients and distribution, while controlling for geographical, life-history and ecological traits. Analyses are conducted primarily at the global scale across all species with elevational data, but also within biogeographic realms, higher taxonomic subsets, and across phylogenetically independent contrasts.

## Materials and Methods

### Datasets and study variables

Two main resources were used in this study: a global species-level database of morphological, life-history, ecological and geographical traits for all known extant bird species [27–29], and a global assessment of avian extinction risk—the International Union for Conservation of Nature (IUCN) Red List (2012.2 update) [30]. Treatment of species follows the standard avian taxonomy of Sibley & Monroe [31], which was updated for newly described species and recent taxonomic revisions. S1 Appendix contains the species-typical data values and sources for each variable and bird species analysed in this study.

#### Response variable: threat of extinction

Our response variable, threat of extinction, used the classifications from the IUCN Red List (2012.2 update). All 9,934 extant bird species recognised by BirdLife International have been fully evaluated under the IUCN Red List categories and criteria [30]. The IUCN Red List status provides the best available comparable estimates of species extinction risk [32]. We scored threat of extinction on a five-point scale: Critically Endangered (CR) = 4, Endangered (EN) = 3, Vulnerable (VU) = 2, Near Threatened (NT) = 1, Least Concern (LC) = 0. Following Bennett & Owens [33], threat of extinction was treated as a continuous variable (see [34] for a discussion on the treatment of the IUCN Red List categories as a linear interval scale). Species which have recently gone extinct (EX; 130 species), are thought to be extinct in the wild (EW; 4 species), or are Data Deficient (DD; 60 species) were excluded from all analyses. Species with an elevational range of zero meters (139 species) were also excluded, in order to focus on those species that possess elevational variation in their geographical range. In addition, these species were removed because: (a) the majority were pelagic seabirds, and (b) some of these species have only been sighted once or a small number of times and as such their true elevational distribution is highly uncertain. Removal of those species with an elevational range of zero metres did not qualitatively influence the findings presented here. A total of 1,239 (13%) of the study-species were listed as ‘Threatened’ (VU, EN or CR), while the vast majority of species (some 78%) are listed as lower risk (LC).

#### Predictors of extinction risk

Full definitions and descriptions of all extinction risk predictors in this study are provided in S2 Table. Our principal extinction risk predictor variables were three measures of elevational distribution—elevational range, maximum elevation and elevational midpoint. The primary source of elevational data was species descriptions from the 16-volume *Handbook of the Birds of the World* [35]. In total, this study includes elevational data for approximately 60% of the world’s extant bird species (S2 Table). Minimum elevation at which a species typically occurs (omitting unconfirmed, predicted, anomalous and extreme outlier records) was excluded as a stand-alone study variable. This was principally due to the large proportion of species with a minimum elevation of approximately zero metres. For a breakdown of predictor variable sample sizes by IUCN Red List category (2012.2 update) and an indication of data completeness, refer to S3 Table. Briefly, this table shows that elevational distribution data is reasonably well represented within each Red List category (i.e. ≥50%), and relatively comparable across Red List categories—although lowest for Critically Endangered species.

Unless specifically stated in the literature, elevational range, over which a species is known to occur, was determined via interpolation as the difference between species-typical maximum and minimum elevational limits. Range interpolation makes the inherent assumption that a species observed at two different elevational levels is present everywhere between these levels, i.e. it assumes continuous species distributions, as is commonly done in ecological studies at all spatial scales (e.g. [36–38]). If minimum and maximum elevational limits were available for different subspecies or regions of a given species’ range, the lowest and highest values across them all were used to calculate elevational range. Elevational range is assumed to represent a proxy measure of competitive ability, propensity to adapt to novel environments, and ability to tolerate environmental variability, as in previous studies (e.g. [39–41]). As summarised by Tobias & Seddon [41], elevational range can be used as a surrogate for both ecological plasticity (i.e. the ability of individuals to adapt from one environment to another or to switch diet types) and ecological generalism (i.e. the ability of individuals to exploit a range of environments simultaneously).

Species-typical maximum elevation excludes unconfirmed, predicted, anomalous and extreme outlier records, and is a parameter constrained by both physiological tolerance (see discussion in [42]) and topography. Elevational midpoint is a proxy measure of central tendency, providing an indication of the mean elevation of a species’ range. Specifically, elevational midpoint was quantified as the mean between species-typical minimum and maximum elevational limits. We did not use interpolation or imputation techniques to obtain elevational data for missing species, as this is not advisable for geographical traits where the majority of variation occurs at the species level [29].

In order to establish the potential strength of elevational distribution as a predictor of extinction risk, we included additional variables, selected based on one or more of the following criteria: (1) data availability and sample size, (2) taxonomic and geographic coverage, and (3) if they have been studied with respect to extinction risk variation in previous studies (for comparative purposes). Specifically, a complementary suite of traits were analysed, reflecting: (a) distribution (geographical range, mean raw latitude, mean absolute latitude), (b) morphology (body weight), (c) reproduction (clutch size, annual fecundity, egg weight), (d) development (incubation period, fledging time, age at first breeding), (e) survival (adult survival), and (f) niche breadth (diet breadth and habitat breadth). Definitions and descriptions of these variables are provided in S2 Table. The detailed protocol followed for data collection and derivation of species-typical values (typically median values) is described in Bennett [27] and White [29]. Most of the extinction risk predictors were log_10_ transformed prior to analysis so that they more closely approximated a normal distribution, except for adult survival, which was arcsine transformed, and raw mean latitude, diet breadth and habitat breadth, which were not transformed.

### Statistical analyses

All data were analysed with the statistical package R v.2.15.1 [43].

#### Bivariate relationships

This study is principally investigating global patterns and the generality of any relationships between extinction risk and potential predictor variables (especially elevational distribution). Our analyses began by using a simple bivariate approach to promote clarity in identifying trends. This approach also maximises statistical power and taxonomic/geographic coverage because it uses the largest possible sample sizes. Reduced Major Axis (RMA) bivariate linear regressions were performed between each of the potential predictors and extinction risk at the global scale across all species. To test for any regional similarities or differences in the global patterns found, bivariate regressions were also conducted for breeding bird species found within each of the biogeographic realms delimited by Olson et al. [44]: Nearctic, Palaearctic, Neotropical, Afrotropical, Indo-Malay, Australasia and Oceania (excluding Antarctica due to small sample sizes). Specifically, regressions within biogeographic realms were conducted for: (a) all breeding species, and (b) breeding endemics only (to investigate the influence of wide-ranging/generalist species). We also investigated the bivariate relationships for species found within the 23 avian orders [31]. Finally, bivariate relationships were tested at the global scale across families. Family-typical values were derived as the mean of constituent generic values, which in turn were calculated as the mean of constituent species values [28]. Bivariate regressions were performed using the ‘lmodel2’ R package [45]

Species are the fundamental units of conservation and also represent the taxonomic level where, using a nested taxonomic model, the greatest level of variation occurs for distributional variables of birds, including all three measures of elevational distribution studied here [29]. Analysis at the family level accounts for the fact that the majority of variation for life-history traits in birds is displayed at the family taxonomic level [27–29]. In addition, repeating analyses at the family level minimises imbalances between samples sizes among variables, while the much reduced samples sizes helps to establish whether any relationships identified at the species level are robust.

#### Multivariate relationships

Stepwise multiple regression models (α-to-enter/remove = 0.05) were performed across species at the global scale, to investigate the relative role of elevational distribution in determining extinction risk, while controlling for potential confounding variables and known correlates of extinction risk. Multiple regressions were performed using the ‘MASS’ R package [46]. Extinction risk was the dependent variable in all models. Elevational range, maximum elevation and elevational midpoint are autocorrelated (elevational range vs. maximum elevation: *r* = 0.85, elevational range vs. elevational midpoint: *r* = 0.72, maximum elevation vs. elevational midpoint: *r* = 0.98; *n =* 5767, *P* = <0.001 [29]). Consequently, each measure of elevational distribution were analysed in separate models. The basal model contained body weight, absolute mean latitude and elevational distribution as predictors. Absolute mean latitude was included as it is a proxy for mean annual temperature—a previously shown predictor of avian threatened species richness, relating to available ambient energy [21]. This basal model was selected in order to investigate if the main potential environmental predictors are correlates. To this basal model, the reproductive and developmental variables with the largest sample sizes, namely clutch size and incubation period, respectively, were entered and removed in turn. This was repeated for adult survival, diet breadth and habitat breadth. From these models (six per measure of elevational distribution), those variables that were significant (α < 0.05) were entered into a final model (one per measure of elevational distribution). To test for the presence of multicollinearity, variance inflation factors (VIFs) were calculated for each model. All VIFs calculated were <5.00, indicating successful minimisation of multicollinearity [47].

Small geographic range size has consistently been shown to be strongly associated with high extinction risk in avian taxa (e.g. [13, 22]). In this study, geographical range was initially included in the basal model, and consistently found to be a strong negative correlate of extinction risk [29]. However, geographical range is used in calculating the IUCN Red List Index [30]. Therefore, any correlation between geographical range and variation in extinction risk is actually confounded due to non-independence [33–34]. Consequently, geographical range was removed as a predictor from all models. It should be noted that a number of studies have sought to resolve this issue of circularity using a variety of methods. For example, by removing species that are threatened due to declines in geographical range (i.e. Criteria B of the Red List; e.g. [22]), or considering threatened species only if they are listed under Criteria A of the Red List (i.e. population reduction; e.g. [48]). However, such approaches not only lead to a reduction in sample size (and consequently statistical power), but geographical range is intrinsically linked (directly or indirectly) to all five of the Red List criteria, e.g. population reduction and small population sizes. For an exploration of this circularity problem, see [49].

#### Phylogenetic independent contrasts

Numerous studies have shown that extinction risk and its correlates are not randomly distributed with respect to phylogeny (see [50]). In order to assess the importance of phylogenetic non-independence, bivariate and multivariate analyses at the global scale were also analysed using phylogenetic independent contrasts (PICs) [51], calculated within the R package ‘caper’ [52], using phylogenetic trees from [53]. Specifically, the first tree using the ‘Ericson backbone’ [54] and ‘Hackett backbone’ [55] were downloaded (http://birdtree.org/) and used. As both trees are based on a calibrated phylogeny, the often applied, yet unrealistic assumption, of equal branch lengths was not necessary. Within ‘caper’, bivariate and multiple linear regressions were conducted using the ‘crunch’ algorithm, which calculates PICs for continuous variables. The ‘caic.robust’ function was used to remove outlying studentised residuals greater than the commonly applied threshold of three which, if retained, may exert undue influence over the obtained results (see [52]). All regressions were forced through the origin. We chose independent contrasts over tree-based methods such as decision trees, because they provide more precise predictions of extinction risk, and, unlike tree-based methods, they deal with pseudoreplication due to phylogenetic non-independence [56].

## Results

### Bivariate relationships

**Fig 1:**
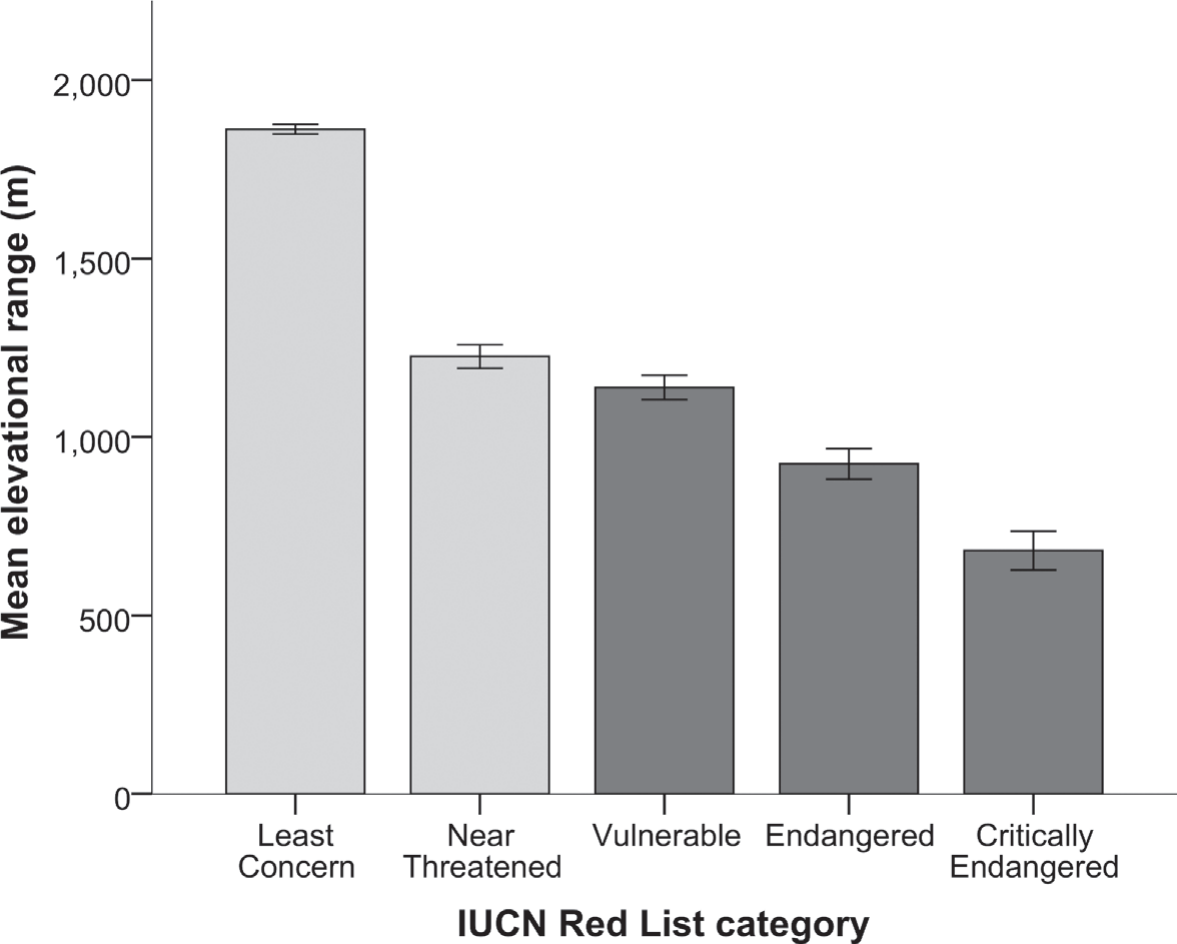
Mean (±1SE) elevational range for bird species with different levels of extinction risk. ANOVA statistics: *n* = 5930 species, *F* = 319.9, *P* = <0.001. Light grey = ‘Not Threatened’ categories of extinction, and dark grey = ‘Threatened’ categories of extinction [30].

**Fig 2:**
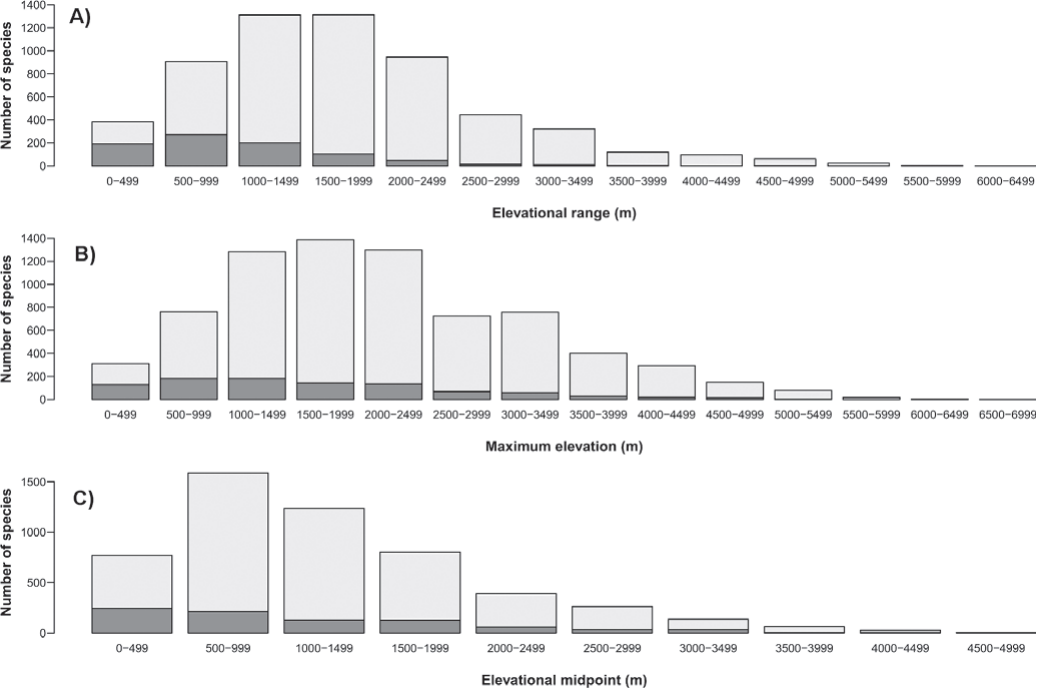
Number of ‘Threatened’ (dark grey: CR, EN, VU) and ‘Not Threatened’ (light grey: LC and NT) bird species [30] with respect to (A) elevational range, (B) maximum elevation, and (C) elevational midpoint. Elevational distribution split into 500 m bands. Due to small samples sizes, it is difficult to establish the number and proportion (%) of ‘Threatened’ species for bands greater than 3500 m, so the values are reported here for clarification. Elevational range: 3500 m = 5 (4%), 4000 m = 1 (1%), 4500 m = 3 (5%), 5000 m = 1 (4%), >5500 m = 0 (0%). Maximum elevation: 3500 m = 28 (7%), 4000 m = 19 (6%), 4500 m = 15 (10%), 5000 m = 3 (4%), >5000 m = 0 (0%). Elevational midpoint: 3500 m = 5 (8%), 4000 m = 4 (17%), 4500 m = 1 (20%).

The three measures of elevational distribution were found to be negatively correlated with extinction risk (Table 1, Fig. 1 and S1 Fig.). Species with narrower elevational ranges, lower maximum elevational limits and lower elevational midpoints are at greater risk of extinction than species with broader and higher elevational distributions. Partitioning species that are ‘Threatened’ (CR, EN and VU) and ‘Not-threatened’ (NT and LC) shows that both number and proportion of ‘Threatened’ bird species decline with increasing elevational distribution (Figs. 2 and S2).

**Table 1:** Pearson correlation coefficients (*r*) between extinction risk and predictors at the global scale across species.

**Predictor group**	**Predictor**	***n***	***r***
**Distribution**	Elevational range	5930	– 0.41***
	Maximum elevation	7464	– 0.26***
	Elevation midpoint	5930	– 0.20***
	Geographical range	9242	– 0.45***
	Raw mean latitude	7505	– 0.03**
	Absolute mean latitude	7505	0.01
**Morphological**	Body weight	8274	0.18***
**Reproduction**	Clutch size	6982	– 0.11***
	Annual fecundity	2215	– 0.26***
	Egg weight	3414	0.30***
**Development**	Incubation period	3055	0.27***
	Fledging time	2637	0.28***
	Age at first breeding	1028	0.29***
**Survival**	Adult survival	447	0.21***
**Niche breadth**	Diet breadth	3435	– 0.01
	Habitat breadth	4030	– 0.30***

* *P* < 0.05

** *P*< 0.01

*** *P* < 0.001. *n* = correlation sample size. Predictors log_10_ transformed except adult survival (arcsine transformed), and raw mean latitude, diet breadth and habitat breadth (untransformed).

Elevational range was the strongest predictor of extinction risk out of the three measures of elevational distribution, and the second strongest (after geographical range) of the 16 variables investigated in total (Table 1). Overall, extinction risk was found to be positively associated with body weight, development and adult survival, but negatively associated with measures of distribution, reproduction and habitat breadth. These relationships for non-elevational predictors support the results of previous broad-scale analyses of extinction risk in birds (e.g. [18, 22, 33, 57–59]). Only absolute mean latitude and diet breadth were not significantly correlated with extinction risk across species at the global scale. Across PICs, all three measures of elevational distribution were still found to be strongly significant negative predictors of extinction risk (S4 Table). Results for non-elevational predictors across PICs were qualitatively similar to those across species, apart from adult survival (no longer significant) and both absolute mean latitude and diet breadth (negatively significant).

Except for Oceanic endemics, the negative relationship between extinction risk and elevational distribution found at the global scale is retained, at a significant level, for species breeding within each biogeographic realm (S5 Table). This negative association is also found when the global assemblage of species is subdivided within the 23 taxonomic orders (S6 Table). Specifically, extinction risk was significantly negatively correlated with elevational range (14 orders), maximum elevation (14 orders) and elevational midpoint (11 orders). Finally, although with reduced significance levels, the negative relationship between extinction risk and elevational distribution found at the global scale across species is also found across families (S7 Table). Results for non-elevational predictors across families were qualitatively similar to those across species, apart from habitat breadth (no longer significant) and diet breadth (positively significant).

### Multivariate relationships

Stepwise multiple regression analysis of the global data, across species, produced models which were qualitatively the same as the outputs from the bivariate tests, but with fewer significant predictors (Tables 2 and S8). Elevational distribution was retained as a significant negative predictor of extinction risk in all models, with elevational range consistently the strongest elevational predictor, followed by maximum elevation and elevational midpoint. In the final model containing elevational range, four of the seven extinction risk predictors were significant; elevational range was the strongest predictor, followed by incubation period, habitat breadth and absolute mean latitude. In the final model containing maximum elevation, five of the seven extinction risk predictors were significant; incubation period was the strongest predictor, followed by maximum elevation, habitat breadth, absolute mean latitude and clutch size. In the final model containing elevational midpoint, five of the seven extinction risk predictors were significant; incubation period was the strongest predictor, closely followed by habitat breadth and elevational midpoint. Both clutch size and absolute mean latitude were less significant. The final models explained 25–31% of the total variance in avian extinction risk. Adult survival was not entered into the final models, due to its lack of (or marginal) significance in the bivariate tests, and the large reductions in sample size its use would entail.

**Table 2:** Multiple regressions of global extinction risk against predictors, across species and phylogenetic independent contrasts (PICs).

	**Predictors**	**Elevational range**			**Maximum elevation**			**Elevational midpoint**		
		**B**	***p***	***r*** ^*2*^	**β**	***p***	***r*** ^*2*^	**β**	***p***	***r*** ^*2*^
**Species**	Elevation	– 0.36	***	0.31	– 0.25	***	0.28	– 0.21	***	0.25
	Body weight	0.001	*NS*		0.01	*NS*		0.01	*NS*	
	Latitude	0.07	*		0.11	***		0.09	**	
	Clutch size	– 0.06	*NS*		– 0.08	**		– 0.09	**	
	Incubation	0.26	***		0.29	***		0.26	***	
	Diet breadth	0.04	*NS*		0.02	*NS*		0.04	*NS*	
	Habitat breadth	– 0.17	***		– 0.18	***		– 0.22	***	
		F_4,823_ = 90.8***			F_5,1017_ = 77.0***			F_5,822_ = 54.7***		
**Ericson**	Elevation	– 0.93	***	0.22	– 0.69	***	0.14	– 0.67	***	0.17
	Body weight	0.14	*NS*		0.07	*NS*		0.14	*NS*	
	Latitude	0.02	*NS*		0.02	*NS*		0.02	*NS*	
	Clutch size	– 0.58	**		– 0.67	***		– 0.92	***	
	Incubation	0.77	**		0.97	***		1.24	***	
	Diet breadth	0.01	*NS*		0.00	*NS*		0.001	*NS*	
	Habitat breadth	– 0.01	*NS*		– 0.002	*NS*		– 0.02	*	
		F_7,802_ = 32.1***			F_7,991_ = 22.5***			F_7,800_ = 23.4***		
**Hackett**	Elevation	– 0.75	***	0.22	– 0.60	***	0.17	– 0.55	***	0.18
	Body weight	0.21	*		0.12	*NS*		0.15	*NS*	
	Latitude	0.09	***		0.07	**		0.11	***	
	Clutch size	– 0.69	***		– 0.81	***		– 0.99	***	
	Incubation	0.74	*		0.86	**		0.91	*	
	Diet breadth	0.01	*NS*		0.00	*NS*		0.001	*NS*	
	Habitat breadth	– 0.01	*NS*		– 0.003	*NS*		– 0.01	*NS*	
		F_7,801_ = 31.9***			F_7,989_ = 28.1***			F_7,800_ = 24.6***		

‘Elevation’ refers to elevational range, maximum elevation and elevational midpoint, respectively, as highlighted at the top of each model column. ‘Latitude’ refers to absolute mean latitude of geographical breeding range. PICs derived from two independent phylogenetic trees, using the ‘Ericson backbone’ and ‘Hackett backbone’. Significance level for a predictor to enter/leave each model was *P* < 0.05. β: multiple regression coefficient (standardised).

* *P* < 0.05

** *P*< 0.01

*** *P* < 0.001. *r* ^*2*^: proportion of variance in extinction risk explained by predictors. *NS*: predictor not retained in model. Degrees of freedom and F-statistic value for each model also reported. Predictors log_10_ transformed, except diet/habitat breadth (untransformed).

Although confounded due to circularity, the inclusion of geographical range size as a predictor made no qualitative difference in the retention of elevational distribution as a significant negative predictor of avian extinction risk (S9 Table). Collectively, elevational distribution and geographical range size explain approximately 24–27% of variation in avian extinction risk.

The three final models were also performed using PICs (Table 2), where elevational distribution remained a strongly significant negative predictor of extinction risk. The other significant predictors that were retained differed according to the measure of elevational distribution entered into the model, and on the phylogeny used to generate PICs. The final models explained 14–22% and 17–22% of the total variance in avian extinction risk using the ‘Ericson backbone’ and ‘Hackett backbone’ phylogenetic trees, respectively.

## Discussion

All three measures of elevational distribution studied here (range, maximum and midpoint) were found to be consistently negatively correlated with avian extinction risk—not just across species globally, but also within biogeographic realms, the majority of taxonomic orders, and across both families and PICs. These findings highlight the importance of elevational distribution as a robust predictor of avian extinction risk (see also [11, 16–19]). Our study is the first to confirm this result comprehensively on a global scale using a large sample, and controlling for potential confounding phylogenetic, environmental and life-history variables.

Our results based on the distribution of current extinction risk highlight the greater vulnerability to extinction of low-elevation bird species. Globally, human impacts, including habitat destruction and overexploitation, predominantly threaten lowland regions at present [14]; see also discussion in Blackburn & Gaston [15]. Although different elevational gradients and mountain ranges worldwide have a unique history of human intervention [14], montane areas remain relatively unspoilt by anthropogenic activities, due largely to their inaccessibility and steep gradients [15]. However, the continued increase in human population levels and natural resource demand has raised concerns that mountain biodiversity is under increasing threat from human pressures, most notably settlement sprawl and agricultural conversion [14, 60–61]. More studies are needed that explicitly investigate the complex and dynamic elevational distribution of different types of anthropogenic threat, at a variety of spatial scales.

The impacts of climate change on extinction risk in mountain regions are complex. We found no evidence for greater current extinction risk in high elevation species than lowland species. This may be because the majority of projected climate change impacts, highlighting the vulnerability of high-montane species (e.g. [11–12]), are predicted to be most severe in the future. In addition, climate change impacts are not currently explicitly incorporated into IUCN Red List assessments of extinction likelihood [30]. Some recent regional studies have shown for birds, and other taxa, that downslope shifts in elevation are just as common as upslope shifts (e.g. [25, 62])—emphasising the taxonomic and spatial heterogeneity of range shifts. Related to this, climate change may also be responsible for higher extinction risk at lower altitudes via several mechanisms, including: 1) the velocity of climate change may be much higher in lowland than montane areas [63], 2) changes in other aspects of climate (e.g. precipitation) may affect lower-elevation species more than temperature changes [25], and 3) lowland biotic attrition [64]. As highlighted in other studies, climate change is combining with ongoing habitat loss and overexploitation in a synergistic manner to pose a growing threat to birds, particularly those on mountaintops, and also those occurring within extensive lowlands with no topographic escape (see [65–66]).

Previous studies that have modelled future extinction risk in montane birds under different scenarios of anthropogenic driven climate and ecosystem change may need modification to account for the lower impact of current extinction filters [67] at high elevations (e.g. [12]). This is because of their relatively intact assemblages of species, including those that may be evolutionarily predisposed to anthropogenic extinction drivers, compared to lowland regions. For example, it has been suggested that species isolated on mountain tops (as with on oceanic islands) might be ecologically naïve [17]. As such, we require more in depth regional investigations and monitoring of the relationships between extinction risk, anthropogenic pressures and elevational distribution in the future.

Although we find evidence suggesting that lowland habitats harbour more threatened species than highlands, threatened species occur across elevational gradients, illustrating the need to expand existing protected areas to protect habitat at all elevations—especially in known mountain biodiversity hotspots [1]. The world’s protected areas are not-randomly distributed [68], with one bias being towards higher elevations as these regions are typically steep, remote, agriculturally unsuitable, and have low human population densities [69]. However, protected area coverage is highly uneven across the world’s mountains and inadequate at a range of scales, including areas of particular importance for biodiversity conservation [70].

Other factors may help explain the negative relationship between extinction risk and elevational distribution. Elsewhere we show that bird species with faster life histories have both larger and higher elevational distributions globally [29]. The relationship between fast life histories and low extinction risk has been found across a range of taxonomic and geographical studies (see [71]), and is supported by the life-history traits analysed in this study, including measures of reproduction and development (Table 1).

Another factor is that lowland continental bird species may be more threatened than montane continental species due to ‘competitive release’ [16, 72], as montane species tend to be relatively common within their restricted ranges, compared to lowland species, and their greater abundance reduces their likelihood of being threatened [17]. Threatened bird species living at higher elevations have also been shown to have larger global population sizes than those occurring in lowlands [15], and consequently may be more resilient to human pressures.

It is important to note that we found both elevational midpoint and maximum elevation were weaker predictors of extinction risk than elevational range. This implies that elevational position has less influence on extinction risk than the breadth of a given species’ elevational range. A large continuous elevational range may provide more places which serve as refuges from anthropogenic impacts, thereby lowering extinction risk [18–19]. An analogous relationship between a species’ geographical range and extinction risk has previously been described (e.g. [13, 16]). In fact, elevational range and geographical range are significantly positively correlated across bird species at the global scale (*r* = 0.43, *n* = 5655, *p* = <0.001 [29]). Nevertheless, we found elevational range to be a strong independent predictor of extinction risk that is complementary to that of geographical range size. Narrow elevational range is therefore not simply a proxy for small planimetric distribution. These two measures of range size are therefore related in terms of how broad a resource base a given species utilises, and both potentially permit a large population size, and act as a buffer against the impacts of habitat loss and human persecution.

Geographical range size is one of the main criteria used to quantify extinction risk and in setting species conservation priorities [73]. Although we appreciate the challenges that such an amendment would entail, our results strongly argue for the addition of elevational distribution into assessments of extinction risk (in agreement with [11, 74]). The relationship between elevational range and extinction risk is largely equivalent in strength to that between geographical range and extinction risk across bird species on a global scale. Some studies have shown that after ‘trimming’ extent of occurrence range maps for birds by their known elevational limits and types of habitat preferred, extents of suitable habitat are often much smaller, especially for species in mountainous regions (e.g. [13, 75]). With considerable advancements in satellite mapping and GIS, such ‘refined extent of occurrence maps’ could be adopted widely. However, currently only a proportion of BirdLife International’s range maps are based in part on elevation [76].

Ultimately, the calculation of three-dimensional (i.e. non-planimetric) range size would provide the most accurate measure of a given species range—especially in montane regions [77]. Non-planimetric range size is a measure of surface area that considers spatial variation in slope. A species that occurs only on a plateau and another species that occurs only on mountainous slopes will therefore possess considerably different surface-area range size, even if their geographical range size is the same. To date, only a handful of ecological studies have calculated and used non-planimetric species range sizes that attempt to merge geographical and elevational distribution into one parameter (e.g. [12]). It would be both informative and innovative to use recent advances in 3D GIS to obtain simplified measures of non-planimetric range sizes for bird species where both geographical and elevational distribution data are available. These values of surface area range size could then be directly compared with geographical range sizes to test for differences and similarities. Furthermore, we need new GIS models that incorporate both habitat structure and elevation distribution to adequately explain global patterns of species richness in terrestrial vertebrates, including the distribution of threatened species.

Our multiple regression analyses account for around a third of the variation in avian extinction risk. This is partly due to the exclusion of extrinsic predictors, particularly those relating to human pressures. Previous studies have found the influence of certain traits on population decline and extinction risk in birds and mammals to be specific to particular threats (e.g. [78–79]). A useful extension to the analyses presented here would be to explore, at a regional scale, the interaction between elevational distribution and direct measures of human impact, such as habitat loss. This would enable a formal investigation into whether or not a given level of impact has a more severe effect on extinction risk of high-elevation or narrow-elevational range species.

The limited influence of body size as a predictor of avian extinction risk in this study is at first surprising, because previous research found it to be a strong positive intrinsic correlate of extinction risk [28, 33]. However, these studies used a subset of the current dataset of around 3,000 species, and since then a large number of small-bodied Neotropical passerines have been added. This greater taxonomic and geographical coverage likely helps to explain the reduced strength of avian body weight as a predictor of extinction risk.

We found that the relationship between elevational distribution and extinction risk is weaker across families than species. This may be influenced by reduced sample sizes and, in turn, statistical power at the family level. However, it may also be because species within families often have a wide range of elevational distributions, with both lowland and montane specialists, as shown by nested models of taxonomic variation in elevational distribution [29].

This study should be extended in the future to include additional predictors. For example, migratory behaviour (which would require a considerable amount of data collection). Migratory behaviour is a well-studied but complex variable, and difficult to incorporate into large-scale interspecific comparative studies such as this. Individual birds within a population may be resident or migrant, and different populations within a species may show varying degrees of migratory movement. Although altitudinal migration is purported to be a common strategy of birds occupying mountainous areas (particularly in the tropics), no extensive literature on the subject exists. Empirical studies documenting the existence and causes of such movement behaviour are scarce and taxonomically and geographically restricted (e.g. [80–81], and references within).

Further work is needed to establish whether our findings are representative of other taxonomic groups (both animal and plants). For example, are the strong and consistent relationships we find for birds, a highly mobile taxa, a general phenomenon characteristic of less-mobile animal groups? Existing evidence seems to suggest that most population declines and disappearances of amphibians have occurred, and are predicted in the future to occur in mid- to high-altitude areas, particularly in the Neotropics (e.g. [82–88])—the opposite to that found here for birds globally. However, elevational distribution data is scarcer than geographical range size data, across taxa. For example, although all known extant mammal species have been assessed under the IUCN Red List categories and criteria [30], elevational distribution data is not currently a data field within the global mammal trait database PanTHERIA [89]. This study highlights the necessity to advance our knowledge of species’ current elevational ranges—information which can be obtained from targeted field excursions and biological collections. We urge the continued collection, collation and utilisation of such data in order to help answer fundamental ecological and conservation-related questions.

## Supporting Information

S1 Appendix Species-typical data values for each study variable and bird species analysed in this study, and bibliography. (XLSX)

S1 Fig Mean (±1SE) elevational distribution (m) for bird species with different levels of extinction risk: (A) maximum elevation and (B) elevational midpoint. ANOVA statistics reported.
(PDF)

S2 Fig Proportion of ‘Threatened’ (CR, EN, VU) bird species with respect to (A) elevational range (*n =* 5930 species), (B) maximum elevation (*n =* 7464 species) and (C) elevational midpoint (*n =* 5930 species). Elevational distribution split into 500 m bands.
(PDF)

S1 Table Summary of studies that have investigated the role of elevational distribution in avian extinction risk. (PDF)

S2 Table Summary of the study predictor variables, including units of measurement, transformation and sample size. (PDF)

S3 Table Predictor sample sizes (*n*) and data completeness by IUCN Red List category for species with data on elevational limits. (PDF)

S4 Table Pearson correlation coefficients (*r*) between extinction risk and predictors at the global scale using phylogenetic independent contrasts (PICs). (PDF)

S5 Table Pearson correlation coefficients (*r*) between extinction risk and elevational distribution for species breeding within individual biogeographic realms (‘All’) and breeding species endemic to individual biogeographic realms (‘Endemic’). Realms are ordered in the table from the strongest to the weakest correlation between elevational range and extinction risk.
(PDF)

S6 Table Pearson correlation coefficients (*r*) between extinction risk and elevational distribution for species within each order. Orders are arranged in the table from the strongest to the weakest correlation between elevational range and extinction risk.
(PDF)

S7 Table Pearson correlation coefficients (*r*) between extinction risk and predictors at the global scale across families. (PDF)

S8 Table Stepwise multiple regressions of extinction risk against predictors at the global scale across species. The table shows the six models used to develop the final model (Table 2).
(PDF)

S9 Table Stepwise multiple regressions of extinction risk against predictors at the global scale across species, with the inclusion of geographical range size. In addition to a ‘simple’ model where only ‘elevation’ and ‘geographical range’ are added as potential predictors, the table shows the final model (Table 2) along with the six models used to develop it.
(PDF)
