# Supplementary material for: Elevational Distribution and Extinction Risk in Birds
Source: PLoS One. 2015 Apr 7;10(4):e0121849. doi: 10.1371/journal.pone.0121849 (PMC4388662; doi:10.1371/journal.pone.0121849)
Supplement: S1 Fig — ANOVA statistics reported. (PDF) [file pone.0121849.s002.pdf]

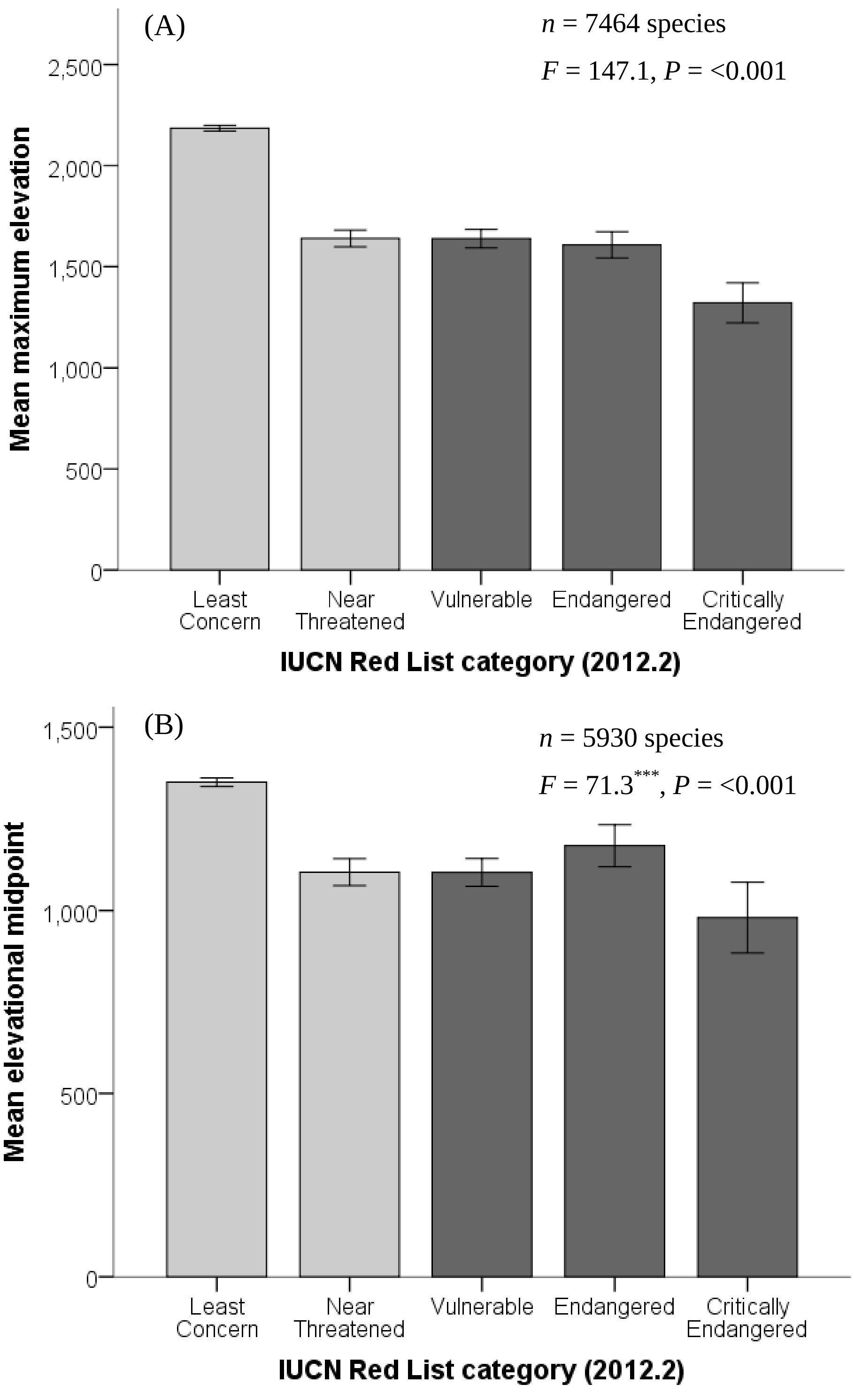

Figure S1. Mean (±1SE) elevational distribution (m) for bird species with different levels of extinction risk: (*a*) maximum elevation and (*b*) elevational midpoint. ANOVA statistics reported.
