# Supplementary material for: Elevational Distribution and Extinction Risk in Birds
Source: PLoS One. 2015 Apr 7;10(4):e0121849. doi: 10.1371/journal.pone.0121849 (PMC4388662; doi:10.1371/journal.pone.0121849)
Supplement: S2 Fig — Elevational distribution split into 500 m bands. (PDF) [file pone.0121849.s003.pdf]

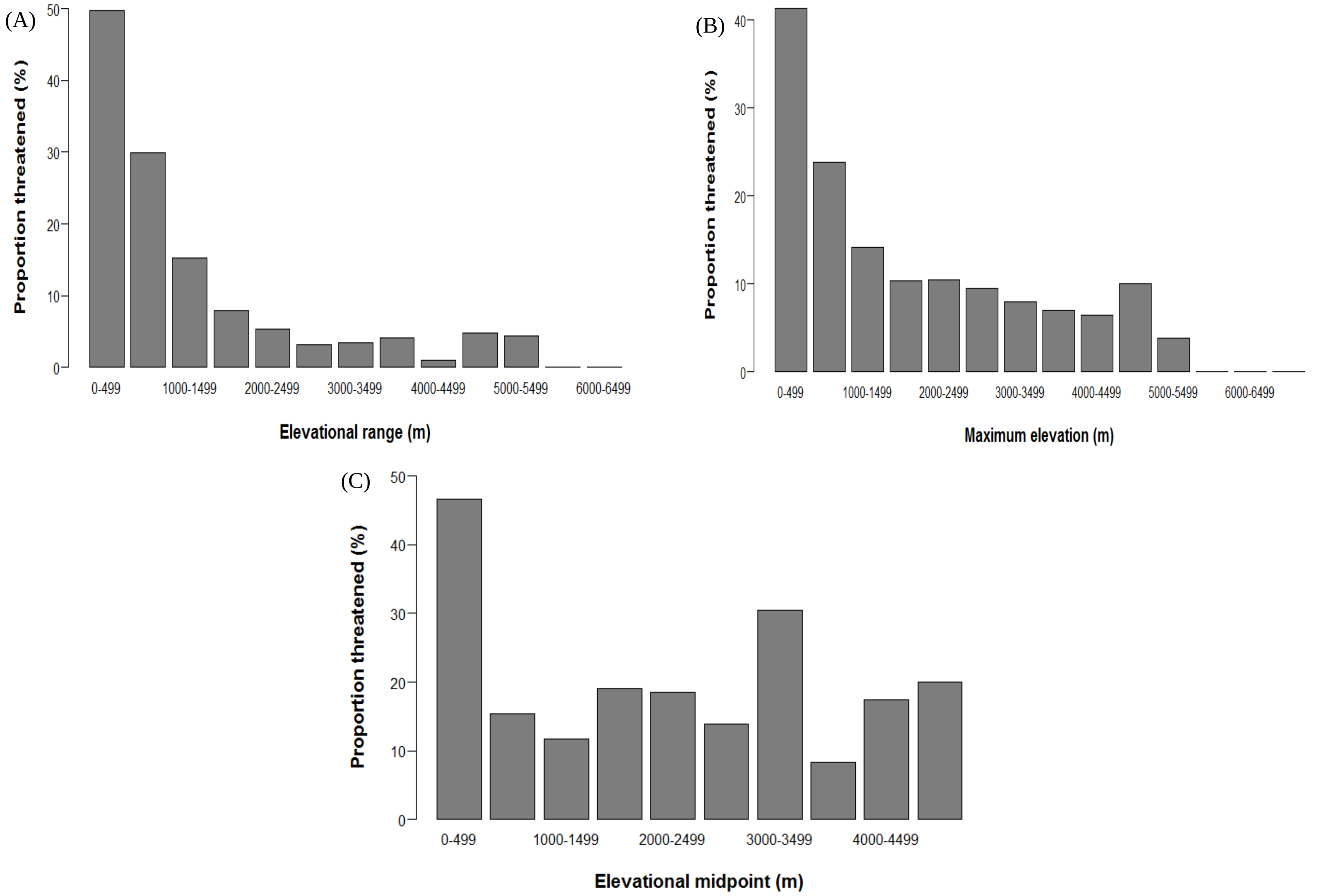


**Figure S2. Proportion of 'Threatened' (CR, EN, VU) bird species with respect to (a) elevational range (*n* = 5930 species), (b) maximum elevation (*n* = 7464 species) and (c) elevational midpoint (*n* = 5930 species)**. Elevational distribution split into 500 m bands.
