# Supplementary material for: Elevational Distribution and Extinction Risk in Birds
Source: PLoS One. 2015 Apr 7;10(4):e0121849. doi: 10.1371/journal.pone.0121849 (PMC4388662; doi:10.1371/journal.pone.0121849)
Supplement: S1 Table — (PDF) [file pone.0121849.s004.pdf]

Table S1. Summary of studies that have investigated the role of elevational distribution in avian extinction risk.

Author (year)	Geographic and taxonomic extent	Main extinction risk and elevation conclusions
**[1] Manne *et al.* (1999)**	American passerines (2286 species, of which 478 were classified as 'montane').	For species with range sizes between 1000 and 100,000 km^2, a much higher proportion of 'lowland' continental than of either 'montane' continental or 'island' species were classified as threatened.
**[2] Manne & Pimm (2001)**	American passerines (2074 species, of which 459 were classified as 'montane').	Species with narrower elevational bands suffer higher levels of threat across lowland, montane and island species. For a given range size, lowland species suffer higher levels of threat than island or montane species.
**[3] Sutherland (2003)**	Global study where country is the main study unit.	Once the total number of species and area are controlled for, there are more endangered birds and mammals in mountainous countries, with the effect size largest for birds.
**[4] Gage *et al.* (2004)**	Approximately half of the known Neotropical bird species (1708 species).	Minimum elevation and elevational range were positively and negatively associated with threat, respectively (raw species analysis), but not for independent contrasts. When species were restricted to a single zoogeographic region elevational range was negatively correlated with threat for both raw and independent contrasts.
**[5] Keane *et al*. (2005)**	Global study of Galliformes (232 out of 284) species	Found elevational range to be negatively associated with extinction risk globally, and when broken down into certain families and regions (raw data and independent contrasts). Elevational (and latitudinal) range found to explain a large proportion of the variance in extinction risk alone.
**[6] Davies *et al*. (2006)**	Global study of landbirds (9626 species), and for six biogeographic realms.	Found elevation range to be a positive predictor of threatened avian species richness globally, and for the Neotropical and Australasian biogeographic realms. The large numbers of zero-sum squares bias results towards a positive correlation between threatened species richness and elevation range.
**[7] Krűger & Radford (2008)**	Global study of 237 species in the family Accipitridae.	No significant relationship was found between median breeding altitude and extinction risk.
**[8] Sekercioglu *et al*. (2008)**	Global study of landbirds (8459 species)	Elevational limitation of range size explained 97% of the variation in the probability of being either 'threatened' or 'at risk'. Species with wider elevational ranges had lower extinction risk.
**[9] Lee & Jetz (2011)**	Global study of landbirds (8664 species)	Both potential minimum elevation and potential elevational range across all species were found to have no association with threat status, and were therefore excluded from the final structural equation models.

1. Manne LL, Brooks TM, Pimm SL (1999) Relative risk of extinction of passerine birds on continents and islands. Nature 399: 258–261.
2. Manne LL, Pimm SL (2001) Beyond eight forms of rarity: which species are threatened and which will be next? Anim Conserv 4: 221–229.
3. Sutherland WJ (2003) Parallel extinction risk and global distribution of languages and species. Nature 423: 276–279.
4. Gage GS, Brooke MD, Symonds MRE, Wege D (2004) Ecological correlates of the threat of extinction in Neotropical bird species. Anim Conserv 7: 161–168.
5. Keane A, Brooke MdL, McGowan PJK (2005) Correlates of extinction risk and hunting pressure in gamebirds (Galliformes). Biol Conserv 126: 216–233.
6. Davies RG, Orme CDL, Olson V, Thomas GH, Ross SG, et al. (2006) Human impacts and the global distribution of extinction risk. Proc R Soc Lond B Biol Sci 273: 2127–2133.
7. Krüger O, Radford AN (2008) Doomed to die? Predicting extinction risk in the true hawks Accipitridae. Anim Conserv 11: 83–91.
8. Sekercioglu CH, Schneider SH, Fay JP, Loarie SR (2008) Climate change, elevational range shifts and bird extinctions. Conserv Biol 22: 140–150.
9. Lee TM, Jetz W (2011) Unravelling the structure of species extinction risk for predictive conservation science. Proc R Soc Lond B Biol Sci 278: 1329–1338.
