# Supplementary material for: Elevational Distribution and Extinction Risk in Birds
Source: PLoS One. 2015 Apr 7;10(4):e0121849. doi: 10.1371/journal.pone.0121849 (PMC4388662; doi:10.1371/journal.pone.0121849)
Supplement: S2 Table — (PDF) [file pone.0121849.s005.pdf]

Table S2. Summary of the study predictor variables, including units of measurement, transformation and sample size. 'Elev. range' and 'Max. elev.' indicate the sample sizes for species with elevational range/midpoint data and maximum elevation data, respectively.

Predictor	Description	Units	Transf.	Sample size	
				Elev. range	Max. elev.
Distribution					
Elevational range	Elevational range over which a species is known to occur. Difference between species-typical maximum and minimum elevational limits.	Metres (m)	Log_{10}	5930	5930
Maximum elevation	Maximum elevation at which a species typically occurs – excluding unconfirmed, predicted, anomalous and extreme outlier records[*].	Metres (m)	Log_{10}	5930	7464
Elevational midpoint	Mean between species-typical minimum and maximum elevational limits.	Metres (m)	Log_{10}	5930	5930
Geographical range	Number of $100km^2$ cells in which a species is known to breed[†].	Count	Log_{10}	5653	7156
Raw mean latitude	Mean of the most northerly and southerly breeding range points. Species whose midpoint is located in the Southern Hemisphere are denoted by negative values.	Degrees (°)	None	4607	5885
Absolute mean latitude	Mean of the most northerly and southerly breeding range points, irrespective of the hemisphere in which it falls.	Degrees (°)	Log_{10}	4607	5885
Morphological					
Body weight	Species-typical adult body weight: preference given to female weight. Where unavailable, species then male weight taken.	Grams (g)	Log_{10}	5110	6477
Reproduction					
Clutch size	Species-typical number of eggs laid in a single nesting[‡].	Count	Log_{10}	4132	5246
Annual fecundity	Species-typical number of eggs produced per year: product of clutch size multiplied by the number of separate broods produced per year[‡].	Count	Log_{10}	1066	1323
Egg weight	Species-typical egg weight.	Grams (g)	Log_{10}	1853	2397

Table S2. Continued.

Predictor	Description	Units	Transf.	Sample size	
				Elev. range	Max. elev.
Development					
Incubation period	Species-typical time taken to incubate a single egg‡§.	Days	Log_{10}	1646	2060
Fledging time	Species-typical time for first individual to fledge. In the vast majority of cases this refers specifically to the elapsed time between hatching and first flying. A minimum time period of seven days was set ‡.	Days	Log_{10}	1375	1750
Age at first breeding	Modal age at which first breeding occurs. Minimum period set at six months to include those species that are known to breed within their first year, but for which no exact data are available.	Months	Log_{10}	491	595
Survival					
Adult survival	Annual survival rate among individuals above the modal age at first breeding, derived from long-term studies of marked bird populations. Values were not included for species threatened with extinction (i.e. Critically Endangered, Endangered, Vulnerable) under the IUCN Red List [1] (version 2012.2) that are either receiving or have recently received considerable conservation action (e.g. extensive predator control, provision of artificial nests/food, captive breeding, translocations/reintroductions).	Percentage (%)	Arcsine	218	262
Niche breadth					
Diet breadth	Species-typical number of food sub-types consumed.	Count	None	2099	2612
Habitat breadth	Species-typical number of distinct habitats used.	Count	None	2498	3077

*More species in the dataset have maximum elevation data than for either elevational range or elevational midpoint. This was due to the fact that the sources used to input data would more often explicitly state maximum elevational limits than minimum elevational limits. †For details of sources and methodology used to produce the polygon breeding range maps and convert them into a grid-cell format from which geographical range was derived, see Orme et al. [2, 3]. ‡Excludes data for obligate brood parasite. §Data for megapodes (Megapodiidae) were excluded because they are mound builders that use geothermal heat to incubate their eggs, and so provide no parental care.

1. IUCN (2001) IUCN Red List Categories and Criteria: Version 3.1. Gland, Switzerland and Cambridge: IUCN Species Survival Commission.
2. Orme CDL, Davies RG, Burgess M, Eigenbrod F, Pickup N, et al. (2005) Global hotspots of species richness are not congruent with endemism or threat. Nature 436: 1016–1019.
3. Orme CDL, Davies RG, Olson VA, Thomas GH, Ding T-S, et al. (2006) Global patterns of geographic range size in birds. PLoS Biology 4: 1276–1283.
