# Supplementary material for: Elevational Distribution and Extinction Risk in Birds
Source: PLoS One. 2015 Apr 7;10(4):e0121849. doi: 10.1371/journal.pone.0121849 (PMC4388662; doi:10.1371/journal.pone.0121849)
Supplement: S3 Table — (PDF) [file pone.0121849.s006.pdf]

Table S3. Predictor sample sizes (*n*) and data completeness by IUCN Red List category for species with data on elevational limits.

	CR		EN		VU		Threatened		NT		LC		Total	
**Predictor**	***n***	**% total**	***n***	**% total**	***n***	**% total**	***n***	**% total**	***n***	**% total**	***n***	**% total**	***n***	**% total**
**Distribution**														
Elevational range	98	49.7	264	67.9	488	67.1	850	64.7	498	56.6	4582	59.7	5930	60.1
Maximum elevation	98	49.7	264	67.9	488	67.1	850	64.7	498	56.6	4582	59.7	5930	60.1
Elevational midpoint	98	49.7	264	67.9	488	67.1	850	64.7	498	56.6	4582	59.7	5930	60.1
Geographical range	81	41.1	233	59.9	450	61.9	764	58.2	469	53.3	4420	57.6	5653	57.3
Raw mean latitude	45	22.8	143	36.8	329	45.3	517	39.4	313	35.6	3777	49.2	4607	46.7
Abs. mean latitude	45	22.8	143	36.8	329	45.3	517	39.4	313	35.6	3777	49.2	4607	46.7
**Morphological**														
Body weight	63	32.0	169	43.4	362	49.8	594	45.2	364	41.4	4152	54.1	5110	51.8
**Reproduction**														
Clutch size	54	27.4	148	38.0	277	38.1	479	36.5	254	28.9	3399	44.3	4132	41.9
Annual fecundity	22	11.2	54	13.9	78	10.7	154	11.7	39	4.4	873	11.4	1066	10.8
Egg weight	21	10.7	50	12.9	122	16.8	193	14.7	84	9.5	1576	20.5	1853	18.8
**Development**														
Incubation period	32	16.2	74	19.0	123	16.9	229	17.4	94	10.7	1323	17.2	1646	16.7
Fledging time	24	12.2	63	16.2	94	12.9	181	13.8	64	7.3	1130	14.7	1375	13.9
Age first breeding	14	7.1	32	8.2	48	6.6	94	7.2	28	3.2	369	4.8	491	5.0
**Survival**														
Adult survival	6	3.0	10	2.6	19	2.6	35	2.7	7	0.8	176	2.3	218	2.2
**Niche breadth**														
Diet breadth	48	24.4	122	31.4	289	39.8	459	35.0	165	18.8	1475	19.2	2099	21.3
Habitat breadth	92	46.7	238	61.2	428	58.9	758	57.7	203	23.1	1537	20.0	2498	25.3

% total = number of species in avian dataset for a given predictor and IUCN Red List category, divided by the total number of species classified under that category (IUCN Red List 2012.2 update). Abs. mean latitude = absolute mean latitude. CR = Critically Endangered, EN = Endangered, VU = Vulnerable, NT = Near Threatened, and LC = Least Concern.
