# Supplementary material for: Elevational Distribution and Extinction Risk in Birds
Source: PLoS One. 2015 Apr 7;10(4):e0121849. doi: 10.1371/journal.pone.0121849 (PMC4388662; doi:10.1371/journal.pone.0121849)
Supplement: S4 Table — (PDF) [file pone.0121849.s007.pdf]

Table S4. Pearson correlation coefficients (*r*) between extinction risk and predictors at the global scale using phylogenetic independent contrasts (PICs). PICs derived from two independent phylogenetic trees, using: (a) 'Ericson backbone' and (b) 'Hackett backbone'.

Predictor	(a) Ericson		(b) Hackett	
	n	***r***	***n***	***r***
**Distribution**				
Elevational range	5817	– 0.44***	5825	– 0.36***
Maximum elevation	7318	– 0.24***	7327	– 0.22***
Elevation midpoint	5807	– 0.17***	5823	– 0.15***
Geographical range	9058	– 0.49***	9039	– 0.49***
Raw mean latitude	7321	0.00	7359	– 0.03**
Absolute mean latitude	7323	0.00	7349	– 0.03**
**Morphological**				
Body weight	8110	0.03**	8126	0.02*
**Reproduction**				
Clutch size	6841	– 0.15***	6831	– 0.09***
Annual fecundity	2162	– 0.17***	2174	– 0.17***
Egg weight	3357	0.09***	3342	0.17***
**Development**				
Incubation period	2988	0.13***	2988	0.10***
Fledging time	2583	0.17***	2574	0.34***
Age at first breeding	1006	0.14***	1007	0.09**
**Survival**				
Adult survival	436	0.06	435	0.05
**Niche breadth**				
Diet breadth	3386	– 0.25***	3396	– 0.23***
Habitat breadth	3949	– 0.31***	3975	– 0.33***

* $P < 0.05$, ** $P < 0.01$, *** $P < 0.001$. n = correlation sample size. Predictors $\log_{10}$ transformed except adult survival (arcsine transformed), and raw mean latitude, diet breadth and habitat breadth (untransformed).
