# Supplementary material for: Elevational Distribution and Extinction Risk in Birds
Source: PLoS One. 2015 Apr 7;10(4):e0121849. doi: 10.1371/journal.pone.0121849 (PMC4388662; doi:10.1371/journal.pone.0121849)
Supplement: S5 Table — Realms are ordered in the table from the strongest to the weakest correlation between elevational range and extinction risk. (PDF) [file pone.0121849.s008.pdf]

Table S5. Pearson correlation coefficients (*r*) between extinction risk and elevational distribution for species breeding within individual biogeographic realms ('All') and breeding species endemic to individual biogeographic realms ('Endemic'). Realms are ordered in the table from the strongest to the weakest correlation between elevational range and extinction risk.

	**elevational range**				**maximum elevation**				**elevational midpoint**			
	All		***Endemic***		***All***		***Endemic***		***All***		***Endemic***	
**Realm**	***n***	***r***	***n***	***r***	***n***	***r***	***n***	***r***	***n***	***r***	***n***	***r***
**Australasia**	985	– 0.48***	782	– 0.47***	1174	– 0.39***	934	– 0.38***	985	– 0.35***	934	– 0.34***
**Indo-Malay**	1012	– 0.48***	527	– 0.45***	1377	– 0.36***	734	– 0.31***	1012	– 0.33***	734	– 0.28***
**Afrotropical**	1002	– 0.46***	875	– 0.47***	1368	– 0.27***	1195	– 0.27***	1002	– 0.18***	1195	– 0.17***
**Oceania**	117	– 0.46***	69	– 0.20	128	– 0.32***	75	– 0.03	117	– 0.23***	75	0.07
**Nearctic**	389	– 0.39***	161	– 0.51***	450	– 0.29***	194	– 0.39***	389	– 0.26***	194	– 0.35***
**Neotropical**	2680	– 0.35***	2475	– 0.35***	3208	– 0.16***	2978	– 0.15***	2680	– 0.11***	2978	– 0.10***
**Palearctic**	793	– 0.28***	288	– 0.41***	1075	– 0.12***	386	– 0.22***	793	– 0.09**	386	– 0.18**

* $P < 0.05$, ** $P < 0.01$, *** $P < 0.001$. *n* = correlation sample size. All three measures of elevational distribution are $\log_{10}$ transformed. Biogeographic realms as delimited by [1].

1. Olson DM, Dinerstein E, Wikramanayake ED, Burgess ND, Powell GVN, et al. (2001) Terrestrial ecoregions of the worlds: a new map of life on Earth. Bioscience 51: 933–938.
