# Supplementary material for: Elevational Distribution and Extinction Risk in Birds
Source: PLoS One. 2015 Apr 7;10(4):e0121849. doi: 10.1371/journal.pone.0121849 (PMC4388662; doi:10.1371/journal.pone.0121849)
Supplement: S6 Table — Orders are arranged in the table from the strongest to the weakest correlation between elevational range and extinction risk. (PDF) [file pone.0121849.s009.pdf]

Table S6. Pearson correlation coefficients (*r*) between extinction risk and elevational distribution for species within each order. Orders are arranged in the table from the strongest to the weakest correlation between elevational range and extinction risk.

	Elevational range		Maximum elevation		Elevational midpoint	
**Avian order**	***n***	***r***	***n***	***r***	***n***	***r***
**Turniciformes**	15	– 0.76**	15	– 0.76**	15	– 0.76**
**Musophagiformes**	21	– 0.56**	22	0.09	21	0.28
**Strigiformes**	197	– 0.54***	221	– 0.41***	197	– 0.30***
**Gruiformes**	107	– 0.53***	116	– 0.42***	107	– 0.42***
**Galliformes**	189	– 0.47***	191	– 0.29***	189	– 0.19**
**Columbiformes**	159	– 0.45***	232	– 0.35***	159	– 0.31***
**Coraciformes**	86	– 0.45***	105	– 0.43***	86	– 0.43***
**Passeriformes**	3685	– 0.43***	4790	– 0.22***	3685	– 0.14***
**Anseriformes**	37	– 0.40*	39	– 0.39*	37	– 0.33*
**Ciconiiformes**	324	– 0.40***	393	– 0.36***	324	– 0.38***
**Craciformes**	65	– 0.40***	65	– 0.31*	65	– 0.24*
**Piciformes**	200	– 0.39***	309	– 0.19***	200	– 0.15*
**Psittaciformes**	297	– 0.30***	308	– 0.17**	297	– 0.09
**Trochiliformes**	298	– 0.23***	302	0.02	298	0.07
**Struthioniformes**	8	– 0.66	10	– 0.65*	8	– 0.66
**Tinamiformes**	22	– 0.27	31	– 0.18	22	– 0.02
**Galbuliformes**	37	– 0.25	44	– 0.15	37	– 0.08
**Cuculiformes**	66	– 0.24	110	– 0.21*	66	– 0.16
**Apodiformes**	59	– 0.21	71	– 0.20	59	– 0.20
**Trogoniformes**	31	– 0.15	38	0.01	31	0.16
**Bucerotiformes**	16	0.03	40	– 0.12	16	0.03
**Coliiformes**	3	*NS*	4	*NS*	3	*NS*
**Upupiformes**	8	*NS*	8	*NS*	8	*NS*

* $P < 0.05$, ** $P < 0.01$, *** $P < 0.001$. *n* = correlation sample size. *NS*: sample size and/or variation too small to calculate correlation coefficient. All three measures of elevational distribution are $\log_{10}$ transformed. The 23 orders follow those recognised by Sibley & Monroe [1].

1. Sibley CG, Monroe BL (1990) Distribution and Taxonomy of Birds of the World. New Haven: Yale University Press.
