# Supplementary material for: Elevational Distribution and Extinction Risk in Birds
Source: PLoS One. 2015 Apr 7;10(4):e0121849. doi: 10.1371/journal.pone.0121849 (PMC4388662; doi:10.1371/journal.pone.0121849)
Supplement: S7 Table — (PDF) [file pone.0121849.s010.pdf]

Table S7. Pearson correlation coefficients (*r*) between extinction risk and predictors at the global scale across families.

Predictor	*n*	*r*
**Distribution**		
Elevational range	140	– 0.20*
Maximum elevation	140	– 0.20**
Elevation midpoint	140	– 0.23**
Geographical range	144	– 0.37***
Raw mean latitude	141	– 0.27**
Absolute mean latitude	141	0.15
**Morphological**		
Body weight	144	0.33***
**Reproduction**		
Clutch size	143	– 0.17*
Annual fecundity	122	– 0.21*
Egg weight	137	0.41***
**Development**		
Incubation period	131	0.33***
Fledging time	125	0.23**
Age at first breeding	100	0.20*
**Survival**		
Adult survival	66	0.25*
**Niche breadth**		
Diet breadth	113	0.41***
Habitat breadth	122	– 0.13

* $P < 0.05$, ** $P < 0.01$, *** $P < 0.001$. *n* = correlation sample size. Predictors $\log_{10}$ transformed except adult survival (arcsine transformed), and raw mean latitude, diet breadth and habitat breadth (untransformed).
