# Supplementary material for: Elevational Distribution and Extinction Risk in Birds
Source: PLoS One. 2015 Apr 7;10(4):e0121849. doi: 10.1371/journal.pone.0121849 (PMC4388662; doi:10.1371/journal.pone.0121849)
Supplement: S8 Table — The table shows the six models used to develop the final model (Table 2). (PDF) [file pone.0121849.s011.pdf]

Table S8. Stepwise multiple regressions of extinction risk against predictors at the global scale across species. The table shows the six models used to develop the final model (Table 2). 'Elevation' refers to elevational range, maximum elevation and elevational midpoint, respectively, as highlighted at the top of each model column. 'Latitude' refers to absolute mean latitude of geographical breeding range.

	**Predictor**	**Elevational range**			**Maximum elevation**			**Elevational midpoint**		
		**β**	***p***	***r²***	**β**	***p***	***r²***	**β**	***p***	***r²***
**Model 1**	Elevation	– 0.32	***	0.15	– 0.20	***	0.09	– 0.15	***	0.07
	Body weight	0.20	***		0.20	***		0.20	***	
	Latitude	0.03	*		0.02	*NS*		– 0.003	*NS*	
		$F_{3,4152} = 239.0^{***}$			$F_{2,5329} = 250.8^{***}$			$F_{2,4153} = 150.0^{***}$		
**Model 2**	Elevation	– 0.28	***	0.16	– 0.19	***	0.12	– 0.16	***	0.11
	Body weight	0.24	***		0.24	***		0.24	***	
	Latitude	0.07	***		0.06	***		0.06	***	
	Clutch size	– 0.09	***		– 0.10	***		– 0.13	***	
		$F_{4,3229} = 150.9^{***}$			$F_{4,4163} = 139.3^{***}$			$F_{4,3229} = 97.0^{***}$		
**Model 3**	Elevation	– 0.33	***	0.21	– 0.23	***	0.17	– 0.20	***	0.14
	Body weight	0.07	*NS*		0.06	*NS*		0.04	*NS*	
	Latitude	0.04	*NS*		0.05	*		0.03	*NS*	
	Incubation	0.25	***		0.29	***		0.28	***	
		$F_{2,1467} = 189.3^{***}$			$F_{3,1855} = 124.6^{***}$			$F_{2,1467} = 120.0^{***}$		
**Model 4**	Elevation	– 0.39	***	0.15	– 0.21	**	0.10	– 0.16	*	0.07
	Body weight	0.02	*NS*		0.06	*NS*		0.05	*NS*	
	Latitude	– 0.12	*NS*		– 0.09	*NS*		– 0.11	*NS*	
	Survival	0.06	*NS*		0.16	*		0.16	*	
		$F_{1,200} = 36.0^{***}$			$F_{2,242} = 12.9^{***}$			$F_{2,199} = 7.9^{***}$		
**Model 5**	Elevation	– 0.40	***	0.22	– 0.27	***	0.15	– 0.20	***	0.10
	Body weight	0.21	***		0.24	***		0.24	***	
	Latitude	0.06	**		0.08	***		0.05	*	
	Diet breadth	– 0.06	**		– 0.11	***		– 0.13	***	
		$F_{4,1789} = 124.4^{***}$			$F_{4,2253} = 96.7^{***}$			$F_{4,1789} = 51.6^{***}$		
**Model 6**	Elevation	– 0.37	***	0.26	– 0.25	***	0.19	– 0.20	***	0.17
	Body weight	0.18	***		0.20	***		0.19	***	
	Latitude	0.10	***		0.12	***		0.09	***	
	Habitat breadth	– 0.19	***		– 0.24	***		– 0.29	***	
		$F_{4,1905} = 165.2^{***}$			$F_{4,2397} = 143.6^{***}$			$F_{4,1905} = 96.6^{***}$		

Significance level for a predictor to enter/leave each model was $P < 0.05$. β: multiple regression coefficient (standardised). * $P < 0.05$, ** $P < 0.01$, *** $P < 0.001$. r^2: proportion of variance in extinction risk explained by predictors. *NS*: predictor not retained in model. Degrees of freedom and F-statistic value for each model also reported. Predictors $\log_{10}$ transformed, except adult survival (arcsine transformed) and diet/habitat breadth (untransformed).
