# Supplementary material for: Elevational Distribution and Extinction Risk in Birds
Source: PLoS One. 2015 Apr 7;10(4):e0121849. doi: 10.1371/journal.pone.0121849 (PMC4388662; doi:10.1371/journal.pone.0121849)
Supplement: S9 Table — In addition to a ‘simple’ model where only ‘elevation’ and ‘geographical range’ are added as potential predictors, the table shows the final model (Table 2) along with the six models used to develop it. (PDF) [file pone.0121849.s012.pdf]

Table S9. Stepwise multiple regressions of extinction risk against predictors at the global scale across species, with the inclusion of geographical range size. In addition to a 'simple' model where only 'elevation' and 'geographical range' are added as potential predictors, the table shows the final model (Table 2) along with the six models used to develop it. 'Elevation' refers to elevational range, maximum elevation and elevational midpoint, respectively, as highlighted at the top of each model column. 'Latitude' refers to absolute mean latitude of geographical breeding range.

	**Predictor**	**Elevational range**			**Maximum elevation**			**Elevational midpoint**		
		**β**	***p***	***r²***	**β**	***p***	***r²***	**β**	***p***	***r²***
**Simple**	Elevation	– 0.24	***	0.27	– 0.16	***	0.24	– 0.17	***	0.25
	Geographical range	– 0.37	***		– 0.43	***		– 0.46	***	
		$F_{2,5650} = 1024.2^{***}$			$F_{2,7153} = 1135.7^{***}$			$F_{2,5650} = 925.3^{***}$		
**Model 1**	Elevation	– 0.18	***	0.26	– 0.14	***	0.24	– 0.14	***	0.25
	Body weight	0.25	***		0.24	***		0.24	***	
	Latitude	0.06	***		0.05	***		0.05	***	
	Geographical range	– 0.37	***		– 0.40	***		– 0.44	***	
		$F_{4,4134} = 363.4^{***}$			$F_{4,5307} = 421.4^{***}$			$F_{4,4134} = 349.4^{***}$		
**Model 2**	Elevation	– 0.16	***	0.26	– 0.13	***	0.17	– 0.13	***	0.26
	Body weight	0.27	***		0.27	***		0.27	***	
	Latitude	0.06	***		0.05	***		0.05	**	
	Clutch size	0.01	*NS*		0.01	*NS*		0.01	*NS*	
	Geographical range	– 0.36	***		– 0.38	***		– 0.41	***	
		$F_{4,3219} = 285.0^{***}$			$F_{4,4151} = 333.8^{***}$			$F_{4,3219} = 278.1^{***}$		
**Model 3**	Elevation	– 0.10	***	0.38	– 0.06	**	0.36	– 0.06	*	0.37
	Body weight	0.13	***		0.14	***		0.13	***	
	Latitude	0.05	**		0.06	**		0.05	*	
	Incubation	0.14	***		0.15	***		0.14	***	
	Geographical range	– 0.48	***		– 0.48	***		– 0.51	***	
		$F_{5,1458} = 176.5^{***}$			$F_{5,1846} = 203.1^{***}$			$F_{5,1458} = 172.8^{***}$		
**Model 4**	Elevation	0.02	*NS*	0.55	0.12	*	0.52	0.09	*NS*	0.55
	Body weight	0.18	***		0.21	***		0.18	*	
	Latitude	– 0.02	*NS*		– 0.01	*NS*		– 0.02	*NS*	
	Survival	– 0.13	*		– 0.09	*NS*		– 0.13	*	
	Geographical range	– 0.76	***		– 0.74	***		– 0.76	***	
		$F_{3,197} = 80.1^{***}$			$F_{3,240} = 87.1^{***}$			$F_{3,197} = 80.1^{***}$		
**Model 5**	Elevation	– 0.17	***	0.40	– 0.15	***	0.39	– 0.17	***	0.40
	Body weight	0.23	***		0.25	***		0.23	***	
	Latitude	0.05	**		0.06	***		0.05	**	
	Diet breadth	– 0.05	*		0.03	*NS*		0.05	*	
	Geographical range	– 0.51	***		– 0.53	***		– 0.51	***	
		$F_{5,1775} = 239.5^{***}$			$F_{4,2237} = 359.2^{***}$			$F_{5,1775} = 239.5^{***}$		

Table S9. Continued.

	Predictor	Elevational range			Maximum elevation			Elevational midpoint		
		**β**	***p***	r^2	**β**	***p***	r^2	**β**	***p***	r^2
**Model 6**	Elevation	– 0.18	***	0.42	– 0.15	***	0.42	– 0.14	***	0.42
	Body weight	0.23	***		0.24	***		0.23	***	
	Latitude	0.08	***		0.09	***		0.07	***	
	Habitat breadth	0.03	*NS*		0.02	*NS*		0.02	*NS*	
	Geographical range	– 0.51	***		– 0.55	***		– 0.58	***	
		$F_{4,1890} = 348.0^{***}$			$F_{4,2379} = 423.6^{***}$			$F_{4,1890} = 341.2^{***}$		
**Final**	Elevation	– 0.13	***	0.49	– 0.09	***	0.47	– 0.10	***	0.49
	Body weight	0.07	*NS*		0.10	*		0.08	*NS*	
	Latitude	0.05	*NS*		0.06	*		0.05	*NS*	
	Clutch size	– 0.003	*NS*		– 0.04	*NS*		– 0.004	*NS*	
	Incubation	0.25	***		0.21	***		0.25	***	
	Diet breadth	0.07	**		0.04	*NS*		0.07	*	
	Habitat breadth	0.01	*NS*		0.03	*NS*		0.01	*NS*	
	Geographical range	– 0.56	***		– 0.55	***		– 0.59	***	
		$F_{4,818} = 194.9^{***}$			$F_{5,1011} = 180.5^{***}$			$F_{4,818} = 192.3^{***}$		

Significance level for a predictor to enter/leave each model was $P < 0.05$. β: multiple regression coefficient (standardised). * $P < 0.05$, ** $P < 0.01$, *** $P < 0.001$. r^2: proportion of variance in extinction risk explained by predictors. *NS*: predictor not retained in model. Degrees of freedom and F-statistic value for each model also reported. Predictors $\log_{10}$ transformed, except adult survival (arcsine transformed) and diet/habitat breadth (untransformed).
